# Astrocytoma in Patients with HIV—Review of the Literature and Case Report

**DOI:** 10.3390/pathogens15030284

**Published:** 2026-03-05

**Authors:** Florentina Dumitrescu, Eugenia-Andreea Marcu, Vlad Pădureanu, Cristiana-Luiza Rădoi-Troacă, Rodica Pădureanu, Lucian Giubelan

**Affiliations:** 1 Department of Infectious Disease, University of Medicine and Pharmacy of Craiova, 200349 Craiova, Romania; florentina.dumitrescu@umfcv.ro (F.D.); luizacristianaradoi@gmail.com (C.-L.R.-T.); lucian.giubelan@umfcv.ro (L.G.); 2 “Victor Babes”, Hospital of Infectious Diseases and Pulmonology from Craiova, 200515 Craiova, Romania; 3 Department of Internal Medicine, University of Medicine and Pharmacy of Craiova, 200349 Craiova, Romania; rodica.padureanu@umfcv.ro

**Keywords:** astrocytoma, IDH wild type, molecular profiling, HIV, multidisciplinary

## Abstract

Astrocytomas are among the most common primary tumors of the central nervous system, arising from astrocytic glial cells and encompassing a wide spectrum of histopathological grades and clinical behaviors. Human immunodeficiency virus (HIV) infection is characterized by chronic immune dysregulation, neuroinflammation, and increased susceptibility to both opportunistic infections and malignancies. The management of astrocytomas in patients living with HIV presents unique clinical challenges but should, whenever feasible, follow standard neuro-oncological principles. We report the case of a 34-year-old man with well-controlled HIV infection who presented in February 2025 with progressive neurological symptoms. Brain imaging revealed a left temporo-insular lesion, and the diagnosis was confirmed by neuronavigation-guided biopsy performed on 31 March 2025. Histopathological and immunohistochemical evaluation established the diagnosis of an isocitrate dehydrogenase (IDH) wild-type astrocytoma, central nervous system (CNS) World Health Organization (WHO) grade 2, according to the 2021 classification of central nervous system tumors.

## 1. Introduction

Gliomas represent the most common group of primary central nervous system (CNS) tumors and comprise a heterogeneous spectrum of neoplasms originating from glial cells. The current classification integrates histopathological and molecular features, with key biomarkers such as isocitrate dehydrogenase (IDH) mutation status, 1p/19q codeletion, ATRX loss, and TP53 mutation playing a central role in diagnosis, prognostic stratification, and therapeutic decision-making. This integrated approach, introduced in the 2021 World Health Organization (WHO) classification of central nervous system tumors, has significantly improved the understanding of glioma biology and enabled more personalized treatment strategies [1].

Astrocytomas are among the most common primary tumors of the CNS, arising from astrocytic glial cells and encompassing a wide spectrum of histopathological grades and clinical behaviors. According to the WHO classification of tumors of the central nervous system, astrocytomas range from low-grade diffuse astrocytomas to highly aggressive glioblastomas, with molecular alterations increasingly guiding diagnosis and prognosis. Despite advances in neuro-oncology, astrocytic tumors remain associated with significant morbidity and mortality, particularly in immunocompromised populations [1].

Human immunodeficiency virus (HIV) infection is characterized by chronic immune dysregulation, neuroinflammation, and increased susceptibility to both opportunistic infections and malignancies [2,3]. While opportunistic infections and primary central nervous system lymphoma remain the most frequent intracranial pathologies in people living with HIV (PLWH), glial tumors such as gliomas have been increasingly reported in recent years. This trend is largely attributed to the widespread use of combination antiretroviral therapy (cART), which has significantly prolonged survival and altered the spectrum of HIV-associated neurological complications. Recent case series and literature reviews suggest that gliomas in HIV-positive patients may present at a younger age compared with the general population and often manifest with nonspecific neurological symptoms, including seizures, focal neurological deficits, and signs of increased intracranial pressure, frequently mimicking opportunistic infections or lymphoma [4].

The relationship between HIV infection and astrocytoma development remains incompletely understood. Proposed mechanisms include chronic immune activation, impaired tumor immune surveillance, direct or indirect effects of viral proteins on glial cells, and the contribution of oncogenic co-infections. Additionally, HIV-associated neuroinflammation and blood–brain barrier dysfunction may create a microenvironment conducive to tumor initiation or progression. However, existing data are limited, largely derived from case reports, small case series, and retrospective studies [5,6,7,8].

Clinical data indicate that immune status at diagnosis, including CD4^+^ T-cell counts and viral load, is highly variable among affected patients, and their prognostic significance remains controversial. Most studies on HIV-positive patients with gliomas are based on small case series or literature reviews. For example, a recent report included 10 patients from a single center, which were combined with previously published cases for a total of 50 patients analyzed for survival outcomes. These studies show substantial heterogeneity in tumor type, WHO grade, treatment regimens, and immune status. Limitations include small sample sizes, retrospective study design, and potential selection bias, which constrain the generalizability of conclusions. These findings underscore the importance of early histopathological diagnosis and standard oncological treatment, as well as the need for increased awareness of gliomas as a differential diagnosis of intracranial lesions in PLWH [4,9].

The management of astrocytomas in patients living with HIV presents unique clinical challenges but should, whenever feasible, follow standard neuro-oncological principles. Current evidence suggests that surgical resection, radiotherapy, and chemotherapy remain the cornerstone of treatment and are generally tolerated in HIV-positive patients, particularly in the era of effective combination antiretroviral therapy [2,4,10]. However, treatment decisions must take into account immune status, potential drug–drug interactions between antiretroviral agents and chemotherapeutics, and the increased risk of treatment-related toxicities and infections [2]. Importantly, delayed diagnosis and empiric treatment for opportunistic infections may negatively impact outcomes, emphasizing the need for early histopathological confirmation. Multidisciplinary management involving neurology, neurosurgery, oncology, and infectious disease specialists is essential to optimize therapeutic strategies and improve clinical outcomes in this complex patient population [2,4].

This study aims to report the clinical, histopathological, and molecular features of a wild-type astrocytoma occurring in a patient with HIV infection and to contextualize this case within the existing literature, with particular emphasis on diagnostic pitfalls, prognostic implications, and management strategies in this clinical setting.

A targeted literature search was conducted in PubMed and Scopus using the terms ‘HIV’, ‘astrocytoma’, ‘glioma’, and ‘non-AIDS defining malignancy’. Articles published in English between 2000 and 2025 were considered. Case reports, case series, and reviews reporting astrocytoma or glioma in HIV-positive patients were included. Studies without molecular or histopathological data were excluded. This methodology aimed to provide a focused overview of current evidence while acknowledging the limitations inherent to narrative reviews.

## 2. Case Report

We present the case of a 34-year-old HIV-positive man admitted in February 2025 to the Infectious Diseases Department of Craiova, Romania, who reported headache, left-sided facial hypoesthesia, and physical asthenia. The symptoms had an insidious onset and progressively worsened over the two months preceding hospitalization.

**Figure 1:**
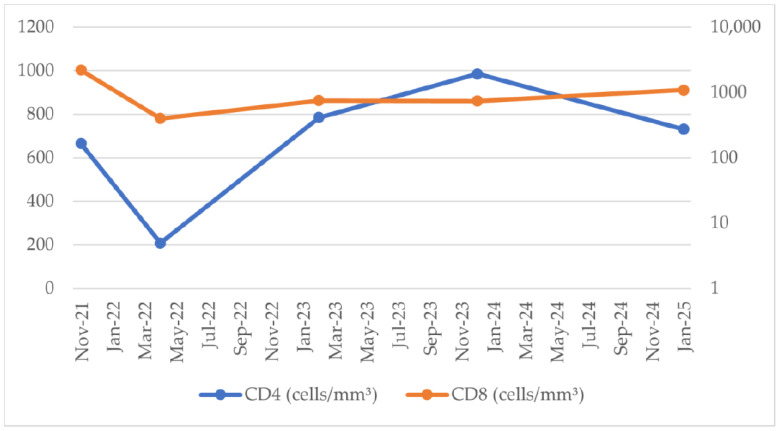
Immunological status.

**Figure 2:**
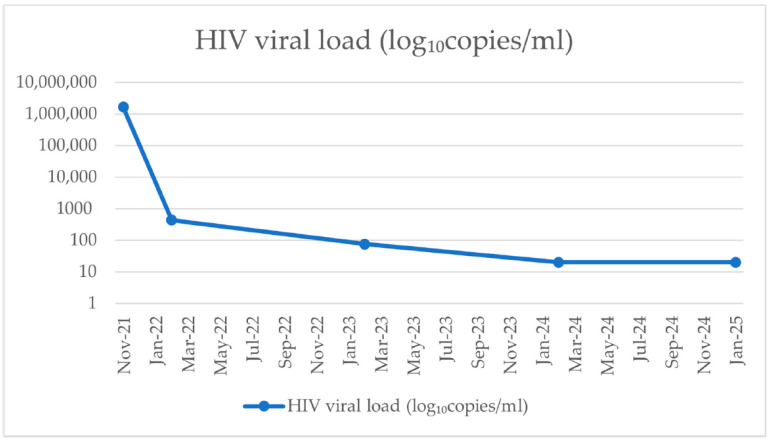
HIV viral load.

The patient’s medical history revealed that he was diagnosed with HIV infection in November 2021, with an initial immunovirological status showing a CD4^+^ T-cell count of 665 cells/mm^3^ and a plasma HIV viral load of 1.66 × 10^6^ copies/mL. Since November 2021, the patient has been on cART consisting of tenofovir disoproxil fumarate, lamivudine, and doravirine (TDF/3TC/DOR), achieving a good immunological and virological response (Figure 1 and Figure 2). His past medical history included persistent oropharyngeal candidiasis and seborrheic dermatitis diagnosed in 2022, as well as latent syphilis identified in January 2025, which was treated with benzathine penicillin G (2.4 million units weekly for three doses). No co-infections with hepatitis B or C viruses were detected, and the patient had no other significant comorbidities.

At admission, the patient was in moderate general condition, normoweight with a body mass index (BMI) of 19.32 kg/m^2^, and presented with stable respiratory and digestive status. Vital signs included blood pressure of 139/66 mmHg, heart rate of 75 beats per minute, and peripheral oxygen saturation (SpO_2_) of 98%. No focal neurological deficits were observed.

Laboratory tests, including complete blood count, hepatic and renal function panels, and inflammatory markers, were within normal limits.

Neurological examination revealed no abnormal postures, neck stiffness, or involuntary movements. Visual acuity was preserved, with intact direct and consensual pupillary light reflexes. Extraocular movements were normal, and pupils were symmetrically reactive. Sensory examination showed left-sided facial hypoesthesia and decreased sensation in the left lateral cervical region, without facial asymmetry or nystagmus. The Romberg test was negative. No dysphagia for liquids or solids was reported. Muscle tone was normal, with preserved coordination on bilateral finger-to-nose testing. Gait was possible without motor deficits. Deep tendon reflexes were symmetrical and normal, except for abolished Achilles tendon reflexes bilaterally. Plantar reflexes elicited bilateral flexion responses. No speech, language, or sensory disturbances were noted, and praxis was preserved.

Neuroimaging studies were performed to further investigate the underlying cause. A cranial CT scan revealed a spontaneously hypodense, poorly defined lesion measuring 36 × 25 mm, located in the left temporal region. The cortical contours were symmetrically preserved bilaterally. The ventricular system was symmetric, of normal size, and midline in position. The pineal gland showed physiological calcification. The frontal, right maxillary, ethmoidal, and sphenoidal sinuses appeared normal on CT. Fluid accumulation was noted in the left maxillary sinus, measuring up to 2.4 cm in thickness, with alveolar recess involvement. Bilateral mastoid pneumatization was normal. Cranial bone structures demonstrated normal morphology.

**Figure 3:**
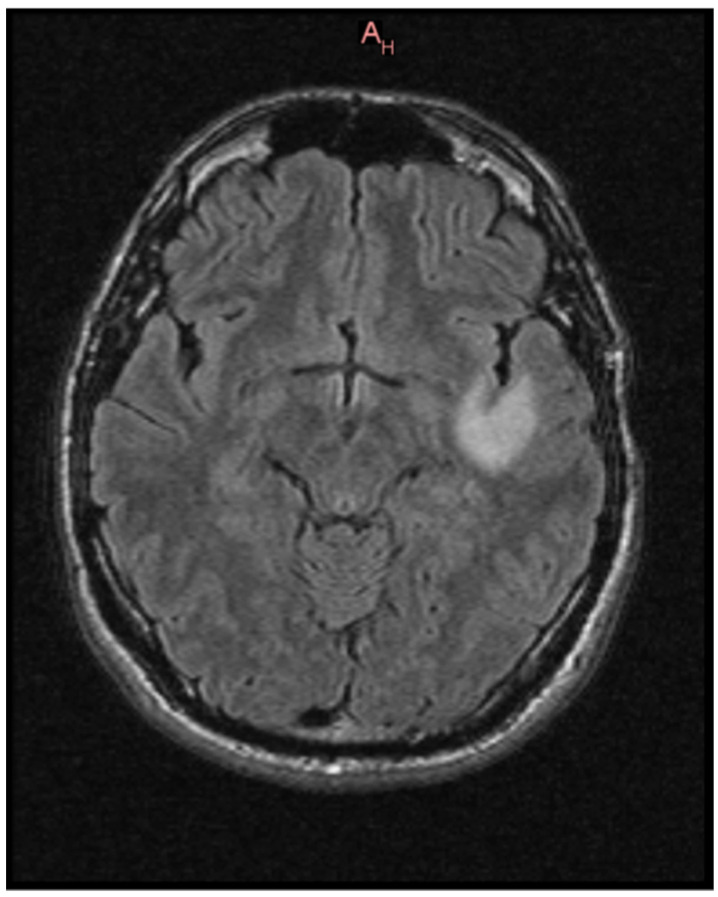
Brain MRI of a left insular–temporal lesion in axial section.

**Figure 4:**
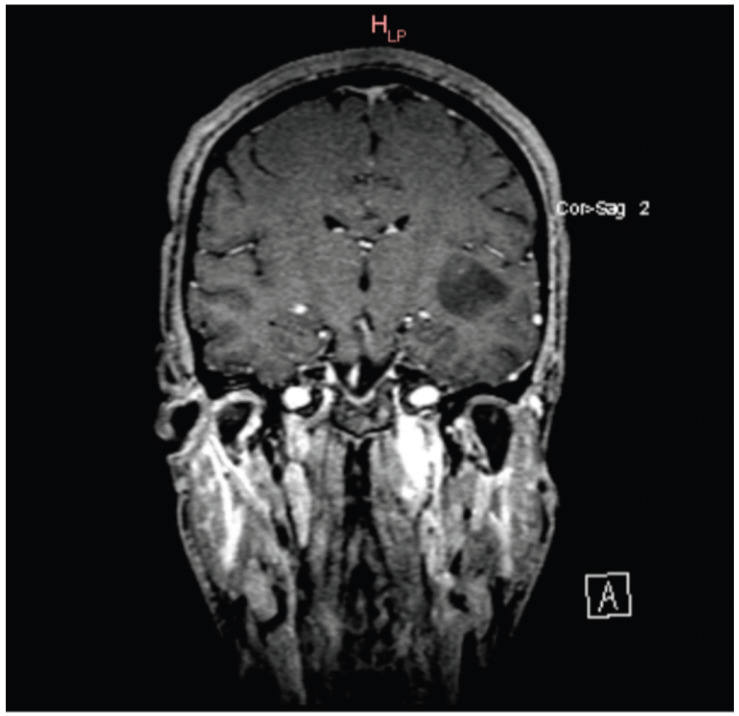
Brain MRI of a left insular–temporal lesion in coronal section.

Subsequent MRI imaging using native and post-contrast conventional sequences revealed:

• A space-occupying lesion measuring 3.7 × 2.8 × 2.3 cm, relatively homogeneous, diffusely infiltrative, located subcortically within the white matter of the left insular and temporal regions. The lesion was hyperintense on T2 and FLAIR sequences, hypointense on T1, with no diffusion restriction on DWI correlated with ADC maps. No contrast enhancement or cerebral edema was associated. These findings suggested a diffuse, low-grade astrocytic glioma, although an oligodendroglioma could not be excluded (Figure 3 and Figure 4).
• Dural thickening up to 7 mm over an approximately 20 × 25 mm area (anteroposterior/cranio-caudal), irregular and vascularized, localized adjacent to the C1 vertebral body, corresponding to spinal nerve roots 1–6 in axial view. The lesion compressed the spinal cord, with a possible “en plaque” meningioma aspect. This dural thickening was partially visible on the current MRI; further cervical spine MRI with contrast was recommended.
• No infra- or supratentorial cerebral lesions suggestive of focal abnormalities, intra- or extra-axial.
• No recent vascular lesions or established sequelae in the vertebrobasilar or carotid territories.
• Ventricular system was symmetric, of normal size, and midline positioned.

A neuronavigation-guided brain biopsy was conducted on 31 March 2025, at Bagdasar-Arseni Clinical Emergency Hospital, Bucharest; no partial or total resection was attempted at this stage. Histopathological and immunohistochemical analyses confirmed a diagnosis of WHO grade 2 left temporo-insular astrocytoma.

Histopathological examination of paraffin-embedded tissue obtained from the left temporo-insular lesion revealed a diffusely infiltrative glial neoplasm with moderately increased cellularity. Tumor cells were medium to large in size, displaying round to oval nuclei with hyperchromatic or euchromatic features, occasional chromocenters, and irregular nuclear contours. Rare mitotic figures were identified (approximately 1 per 10 high-power fields), and no areas of necrosis, microvascular proliferation, or dystrophic microcalcifications were observed. Focal intratumoral hemorrhage and perivascular aggregation of tumor cells were present, with occasional satellitosis around entrapped neurons. Scattered gemistocytic-appearing cells with abundant eosinophilic cytoplasm and eccentrically displaced nuclei were also noted.

Immunohistochemical analysis demonstrated negative staining for the IDH1 R132H mutation, with preserved internal control, and diffuse nuclear positivity for OLIG2. The Ki-67 (MIB-1) labeling index was approximately 5%, showing heterogeneous expression. Loss of ATRX expression was observed in tumor cells, while p53 immunostaining revealed strong and moderate nuclear positivity in approximately 75% of cells, suggestive of a mutant TP53 phenotype. H3K27me3 expression was retained, and CD34 immunostaining was negative in tumor cells, with no evidence of microvascular proliferation. Overall, the histopathological and immunohistochemical findings were consistent with a diagnosis of astrocytoma, IDH wild type, CNS WHO grade 2, according to the 2021 WHO Classification of Tumors of the Central Nervous System [1].

At the follow-up brain MRI performed four months after the initial examination in March 2025, a comparative evaluation revealed persistence of the space-occupying lesion in the left temporo-insular region, with a slight decrease in size measuring 3.6 × 2.85 × 2.5 cm compared to 3.7 × 2.8 × 2.3 cm previously. The lesion remains diffusely infiltrative without signs of cerebral edema or contrast enhancement, but with susceptibility artifacts consistent with post-biopsy hemorrhage. The previously described dural thickening has slightly increased to 8 mm, located adjacent to the C1 vertebral body, compressing the spinal cord, with imaging features suggestive of an “en plaque” meningioma. Further MRI examination of the cervical spine with contrast is recommended.

Following multidisciplinary evaluation, surgical resection followed by adjuvant radiotherapy was recommended in accordance with standard neuro-oncological guidelines. The patient, after being informed about the potential risks, benefits, and prognostic implications, declined both neurosurgical intervention and radiotherapy and opted for a conservative management strategy. Consequently, a program of close clinical and radiological surveillance was initiated.

## 3. Discussion

The coexistence of HIV infection and primary brain tumors such as astrocytomas is uncommon, and the underlying relationship remains incompletely understood. As cART has extended the life expectancy of people living with HIV, the relative incidence of non-AIDS-defining malignancies, including various solid tumors and gliomas, has increased. Although certain cancers such as Kaposi sarcoma, non-Hodgkin lymphoma, and cervical cancer are well-established in the context of HIV, the occurrence of gliomas has been reported increasingly, albeit still at low absolute numbers [11,12].

Epidemiological data from a large case series and literature review suggest that cerebral gliomas, including astrocytomas and glioblastomas, may occur more frequently in HIV-positive populations than previously recognized. In a pooled analysis including 50 HIV-positive patients with glioma, the median overall survival was approximately 9 months, and most deaths were attributed to tumor progression rather than HIV-related complications. Importantly, radiotherapy and WHO tumor grade emerged as independent predictors of survival, whereas immune status (CD4^+^ count) and use of antiretroviral therapy showed trends toward improved survival without achieving statistical significance [4].

Our case highlights several important clinical aspects. First, the patient presented with neurological symptoms consistent with a left temporo-insular lesion, which was confirmed by neuroimaging and biopsy to be a WHO grade 2 astrocytoma, IDH wild type.

The tumor was classified as a WHO grade 2 IDH wild-type astrocytoma based on histopathology, ATRX loss, and TP53 mutation. Additional molecular markers recommended for comprehensive stratification under the 2021 WHO framework, including TERT promoter mutation, EGFR amplification, and chromosome 7/10 status, were not assessed due to specimen limitations. Therefore, molecular glioblastoma features cannot be fully excluded, representing a limitation of the molecular characterization in this case. Future studies should incorporate comprehensive molecular profiling to improve diagnostic precision in HIV-positive patients presenting with diffuse astrocytomas.

The patient’s well-controlled HIV infection, evidenced by stable CD4 counts and suppressed viral load on combination antiretroviral therapy, suggests that tumor development occurred despite effective HIV management.

Molecular characterization and histopathology

The histopathological findings, including negative IDH1 mutation status, ATRX loss, and mutant p53 phenotype, support the diagnosis of a low-grade astrocytoma with a potentially more aggressive molecular profile. This highlights the importance of comprehensive molecular characterization in guiding prognosis and treatment decisions.

The molecular profile observed in our patient warrants further discussion. IDH wild-type astrocytomas, regardless of histological grade, exhibit distinct molecular signatures compared to IDH mutant tumors and are generally associated with a more aggressive clinical course. According to the 2021 World Health Organization Classification of Tumors of the Central Nervous System, integrated molecular analysis has become essential in the diagnosis of adult diffuse gliomas, as key biomarkers such as IDH mutation status, ATRX expression, and TP53 alterations provide important prognostic information and guide clinical decision-making [1,13].

TP53 mutations are among the most frequent genetic alterations in astrocytic tumors and play a central role in gliomagenesis by promoting genomic instability and resistance to apoptosis. Loss of ATRX expression is associated with alternative lengthening of telomeres (ALT), a telomere maintenance mechanism commonly observed in astrocytomas and linked to tumor progression. The coexistence of TP53 mutation and ATRX loss, as observed in our case, is characteristic of astrocytic lineage tumors and further supports the diagnosis of diffuse astrocytoma with potentially unfavorable biological behavior [14,15,16].

Furthermore, IDH wild-type diffuse astrocytomas frequently harbor additional molecular alterations, such as TERT promoter mutations, EGFR amplification, and PTEN pathway abnormalities, which are associated with poorer prognosis and molecular overlap with glioblastoma. Therefore, comprehensive molecular profiling is increasingly recommended to improve risk stratification and therapeutic planning in these patients [1,14,17].

Accumulating evidence indicates that molecular markers have significant prognostic implications beyond conventional histopathological grading. IDH wild-type diffuse astrocytomas frequently harbor additional genetic alterations, including TERT promoter mutations, EGFR amplification, and PTEN pathway abnormalities, which are associated with poorer outcomes and molecular overlap with glioblastoma. Consequently, comprehensive molecular profiling is crucial for accurate risk stratification, prognostication, and therapeutic decision-making, particularly in patients with IDH wild-type tumors [13,18].

Pathogenetic mechanisms linking HIV infection and glioma development

The mechanisms linking HIV infection and the development of astrocytic tumors remain incompletely understood, but several biologically plausible pathways have been proposed. Chronic HIV-associated neuroinflammation and persistent immune activation may alter the central nervous system microenvironment, potentially facilitating oncogenic processes. HIV infection induces microglial activation and the release of pro-inflammatory cytokines, creating a neuroinflammatory milieu that could support tumor initiation and progression. Viral proteins such as gp120 and Tat have been shown in experimental models to influence glioma cell proliferation, apoptosis, and metabolic activity, suggesting indirect oncogenic effects of viral components on glial cells [2,4].

Although biologically plausible mechanisms link HIV infection to glioma development—including chronic neuroinflammation, immune dysregulation, and viral protein effects—current evidence is limited and largely derived from small case series or individual reports. No direct causal relationship between HIV infection, ART, and astrocytoma formation has been established, and the potential contribution of HIV should be interpreted cautiously within the broader context of tumor biology and patient-specific factors [2,4,19,20].

In PLWH, cART improves immune function and reduces opportunistic infections but does not fully reverse chronic immune activation. Residual inflammation and immune dysregulation may persist despite long-term viral suppression, contributing to oxidative stress and impaired tumor immune surveillance [19,20]. These mechanisms may promote an environment permissive to tumorigenesis and could partly explain the increasing incidence of non-AIDS-defining malignancies in the cART era [21]. However, current evidence remains limited and largely derived from case reports or small retrospective studies, and no causal relationship between HIV infection, specific ART regimens, and astrocytoma development has been clearly established. Further prospective and molecular studies are required to better define the pathogenetic pathways and clinical implications in this population.

Case reports and small series also highlight the diagnostic challenges in this population. Central nervous system lesions in PLWH are more frequently attributed to opportunistic infections or lymphoma, which may delay consideration of primary brain tumors in differential diagnoses. Brainstem gliomas and other rare tumor presentations have been documented, underscoring the need for thorough imaging and tissue diagnosis when clinical signs persist or progress despite empiric treatment.

Management of gliomas in HIV-positive patients generally follows standard neuro-oncology principles, including maximal safe surgical resection, radiotherapy, and, when appropriate, chemotherapy. A retrospective series of HIV-positive patients with glioblastoma demonstrated that multimodal treatment regimens similar to those used in the general population were well tolerated and associated with survival outcomes primarily driven by tumor biology rather than HIV status [10]. Given the potential for drug–drug interactions between certain chemotherapeutic agents and antiretroviral medications, careful coordination of care is essential. Emerging therapies, such as immune checkpoint inhibitors, have shown promise in case reports, suggesting that immunotherapy may be feasible and safe in select HIV-positive patients with gliomas, though more data are needed [22].

Clinical outcomes and treatment considerations in HIV-positive versus HIV-negative glioma patients

Comparative data between HIV-positive and HIV-negative glioma patients remain limited and heterogeneous. Available evidence suggests that overall survival is largely determined by tumor-related factors, such as WHO grade and receipt of radiotherapy, rather than HIV status itself. In a pooled analysis including 50 HIV-positive patients and a matched cohort of HIV-negative glioma cases, median overall survival was approximately 9 months in both groups [4,19]. Immunological differences were observed, with HIV-positive patients displaying higher CD163^+^ macrophage infiltration and variable CD4^+^ T-cell counts; in some reports, higher CD4^+^ counts and receipt of effective antiretroviral therapy correlated with longer survival, although these associations did not reach statistical significance [19,21]. Reviews of HIV-associated glioblastoma indicate that tumor progression, rather than AIDS-related complications, predominantly dictates outcomes, and standard neuro-oncological treatments—including surgery, radiotherapy, and chemotherapy—appear feasible and generally well tolerated in HIV-positive patients, without unexpected toxicities [2,20]. However, definitive conclusions regarding differences in treatment toxicity, immune considerations, and long-term outcomes between HIV-positive and HIV-negative glioma patients cannot be drawn due to small sample sizes, retrospective designs, and the paucity of prospective comparative studies. These findings highlight the need for larger, multi-institutional cohorts and prospective investigations to better understand the impact of HIV infection and antiretroviral therapy on glioma biology and clinical outcomes.

Radiotherapy remains a cornerstone in the management of low-grade gliomas, especially when surgical resection is limited or not feasible. Our patient has recently initiated radiotherapy, with early MRI follow-up showing stable or slightly reduced tumor size, suggesting a favorable initial response.

Our findings support existing data suggesting that tumor-related factors, including histopathological grade and molecular profile, are likely more relevant to prognosis than HIV-related parameters such as CD4^+^ T-cell count or viral load. The presence of molecular features associated with a potentially more aggressive behavior underscores the importance of comprehensive histopathological and molecular evaluation in HIV-positive patients presenting with intracranial lesions.

Compared with previously reported cases, which highlighted the rarity and marked clinical heterogeneity of gliomas in PLWH, the present case provides additional clinical and molecular insights. Most available reports describe predominantly high-grade tumors, particularly glioblastoma, often in patients with advanced or poorly controlled infection [4]. In contrast, our patient presented with well-controlled infection, preserved immune function, and sustained viral suppression, supporting the hypothesis that glioma development in this population may occur independently of severe immunosuppression. Similar observations have been reported in recent reviews, which emphasize the limited number of published cases and the variability in clinical presentation, treatment, and survival outcomes [4,8,10,22] (Table 1).

Furthermore, the present case highlights the importance of early neuroimaging and histopathological confirmation in patients with nonspecific neurological symptoms, even in the absence of significant immune deterioration. The detailed molecular characterization, including ATRX loss and TP53 mutation in IDH wild-type astrocytoma, contributes to the growing body of evidence suggesting that tumor-related molecular features, rather than infection-related parameters, may play a central role in prognosis [1,13,14,15,16]. Emerging data also indicate that the molecular landscape and treatment response of gliomas in immunocompromised patients may differ from those in the general population, supporting the need for individualized therapeutic strategies [2,10,22].

Additionally, this case illustrates real-world clinical challenges related to treatment decision-making and patient preferences, which remain insufficiently addressed in the current literature [4,19,21]. To our knowledge, detailed molecular characterization of low-grade IDH wild-type astrocytomas in patients with well-controlled HIV infection remains rarely reported. Therefore, our findings support the need for further multicenter studies focusing on molecular profiling, treatment tolerance, and long-term survival in this population.

This study has several limitations. First, as a single case presentation, the findings cannot be generalized to the broader population of PLWH and glioma. Second, although a narrative review of the literature was performed, the available data are limited by small sample sizes, retrospective designs, and marked clinical heterogeneity, as previously highlighted by published case series. Third, long-term follow-up data are not yet available, which limits conclusions regarding survival and treatment response.

Additionally, despite comprehensive histopathological and molecular characterization, advanced genomic sequencing techniques were not performed, which may further refine prognostic stratification in future studies. Therefore, larger multicenter studies with standardized molecular profiling and prospective follow-up are needed to better understand the interaction between human immunodeficiency virus infection and glioma biology.

This single-case report inherently limits generalizability, and while it highlights potential pathogenetic mechanisms, the findings should be interpreted cautiously. Larger, prospective studies are needed to clarify the impact of HIV infection and cART on glioma biology and patient outcomes.

## 4. Conclusions

Astrocytomas in people living with HIV are rare and insufficiently characterized. This case illustrates the occurrence of a WHO grade 2, IDH wild-type astrocytoma in a young patient with well-controlled HIV infection, highlighting that such tumors may develop despite effective antiretroviral therapy. These findings support the concept that tumor-related factors and molecular characteristics may have a greater impact on prognosis than HIV-related parameters.

Early recognition of gliomas as a differential diagnosis of intracranial lesions in people living with HIV is essential to prevent diagnostic delay. Histopathological confirmation and molecular profiling remain crucial for accurate diagnosis and prognostic assessment. Standard neuro-oncological treatments appear feasible in selected HIV-positive patients, emphasizing the importance of multidisciplinary management and close clinical and radiological follow-up.

## Figures and Tables

**Table 1:** Reported gliomas in PLWH: clinical and molecular characteristics.

Author, Year	Number of Patients	Age	Immune Status	Tumor Type	Molecular Data	Treatment	Outcome
Xiao Ding et al., 2024 [4]	50	23–57	Variable CD4	Gliomas	Limited	Surgery ± radiotherapy	~9 months
Maria J. Mendez Valdez et al., 2022 [8]	Review	Not available	Not available	Glioblastoma predominant	Rarely reported	Multimodal	~8 months
James R. Hall et al., 2009 [10]	21	19–60	Variable CD4 (80–610 cells/mm^3^)	Glioblastoma	Not reported	Radiotherapy + chemotherapy	~8 months
Christine A. Yuen et al., 2024 [22]	1	39	Controlled HIV	Glioblastoma	Molecular data	Immunotherapy	Clinical benefit
Present case	1	34	Preserved CD4, suppressed viral load	Astrocytoma grade 2	Detailed molecular profile	Surveillance	Stable

## Data Availability

The original contributions presented in this study are included in the article. Further inquiries can be directed to the corresponding authors.

## References

[1] Louis D.N., Perry A., Wesseling P., Brat D.J., Cree I.A., Figarella-Branger D., Hawkins C., Ng H.K., Pfister S.M., Reifenberger G. (2021). The 2021 WHO Classification of Tumors of the Central Nervous System: A Summary. Neuro-Oncology.

[2] Deeken J.F., Tjen-A-Looi A., Rudek M.A., Okuliar C., Young M., Little R.F., Dezube B.J. (2012). The Rising Challenge of Non-AIDS-Defining Cancers in HIV-Infected Patients. Clin. Infect. Dis..

[3] Barczak S., Badura B., Lembas A., Mikuła T., Wiercińska-Drapało A. (2024). Severe Course of HIV-Related Kaposi’s Sarcoma with Cutaneous, Visceral and Oral Manifestations in a Late-Presenting Patient. Prospect. Pharm. Sci..

[4] Ding X., Liang T., Liang B., Zhou X., Chen J., Gao H., Wang F., Zheng X., Feng E. (2024). Clinical Characteristics and Prognostic Analysis of Patients with HIV and Glioma: A Case Series and Literature Review. Exp. Ther. Med..

[5] Eugenin E.A., Clements J.E., Zink M.C., Berman J.W. (2011). Human Immunodeficiency Virus Infection of Human Astrocytes Disrupts Blood-Brain Barrier Integrity by a Gap Junction-Dependent Mechanism. J. Neurosci..

[6] Hong S., Banks W.A. (2015). Role of the Immune System in HIV-Associated Neuroinflammation and Neurocognitive Implications. Brain Behav. Immun..

[7] Torices S., Daire L., Simon S., Naranjo O., Mendoza L., Teglas T., Fattakhov N., Adesse D., Toborek M. (2023). Occludin: A Gatekeeper of Brain Infection by HIV-1. Fluids Barriers CNS.

[8] Mendez Valdez M.J., Lu V.M., Kim E., Rivas S.R., Govindarajan V., Ivan M., Komotar R., Nath A., Heiss J.D., Shah A.H. (2022). Glioblastoma Multiforme in Patients with Human Immunodeficiency Virus: An Integrated Review and Analysis. J. Neurooncol..

[9] Wang T., Gao T., Niu X., Xing X., Yang Y., Liu Y., Mao Q. (2018). Clinical Characteristics and Prognostic Analysis of Glioma in Human Immunodeficiency Virus-Infected Patients. World Neurosurg..

[10] Hall J.R., Short S.C. (2009). Management of Glioblastoma Multiforme in HIV Patients: A Case Series and Review of Published Studies. Clin. Oncol. (R. Coll. Radiol.).

[11] Lim S.T., Levine A.M. (2005). Non-AIDS-Defining Cancers and HIV Infection. Curr. HIV/AIDS Rep..

[12] HIV Infection and Cancer Risk—NCI. https://www.cancer.gov/about-cancer/causes-prevention/risk/infectious-agents/hiv-fact-sheet.

[13] Brat D.J., Aldape K., Bridge J.A., Canoll P., Colman H., Hameed M.R., Harris B.T., Hattab E.M., Huse J.T., Jenkins R.B. (2022). Molecular Biomarker Testing for the Diagnosis of Diffuse Gliomas: Guideline from the College of American Pathologists in Collaboration with the American Association of Neuropathologists, Association for Molecular Pathology, and Society for Neuro-Oncology. Arch. Pathol. Lab. Med..

[14] Brat D.J., Verhaak R.G.W., Aldape K.D., Yung W.K.A., Salama S.R., Cooper L.A.D., Rheinbay E., Miller C.R., Vitucci M., Cancer Genome Atlas Research Network (2015). Comprehensive, Integrative Genomic Analysis of Diffuse Lower-Grade Gliomas. N. Engl. J. Med..

[15] Ohgaki H., Kleihues P. (2007). Genetic Pathways to Primary and Secondary Glioblastoma. Am. J. Pathol..

[16] Chaudhry N.S., Ahmad F.U., Blieden C., Benveniste R.J. (2013). Brainstem Anaplastic Glioma in Patients with AIDS: A Case Report and Review of the Literature. BMJ Case Rep..

[17] Eckel-Passow J.E., Lachance D.H., Molinaro A.M., Walsh K.M., Decker P.A., Sicotte H., Pekmezci M., Rice T., Kosel M.L., Smirnov I.V. (2015). Glioma Groups Based on 1p/19q, IDH, and TERT Promoter Mutations in Tumors. N. Engl. J. Med..

[18] Brat D.J., Aldape K., Colman H., Holland E.C., Louis D.N., Jenkins R.B., Kleinschmidt-DeMasters B.K., Perry A., Reifenberger G., Stupp R. (2018). cIMPACT-NOW Update 3: Recommended Diagnostic Criteria for “Diffuse Astrocytic Glioma, IDH-Wildtype, with Molecular Features of Glioblastoma, WHO Grade IV”. Acta Neuropathol..

[19] Silverberg M.J., Chao C., Leyden W.A., Xu L., Tang B., Horberg M.A., Klein D., Quesenberry C.P., Towner W.J., Abrams D.I. (2009). HIV infection and the risk of cancers with and without a known infectious cause. AIDS.

[20] Hleyhel M., Hleyhel M., Bouvier A.M., Belot A., Tattevin P., Pacanowski J., Genet P., De Castro N., Berger J.-L., Dupont C. (2014). Risk of Non-AIDS-Defining Cancers among HIV-1-Infected Individuals in France between 1997 and 2009: Results from a French Cohort. AIDS.

[21] Shiels M.S., Pfeiffer R.M., Gail M.H., Hall H.I., Li J., Chaturvedi A.K., Bhatia K., Uldrick T.S., Yarchoan R., Goedert J.J. (2011). Cancer Burden in the HIV-Infected Population in the United States. J. Natl. Cancer Inst..

[22] Yuen C.A., Bao S., Pekmezci M., Mo F., Kong X.-T. (2024). Pembrolizumab in an HIV-Infected Patient with Glioblastoma. Immunotherapy.

